# Supplementary material for: Identifying children who develop severe chronic kidney disease using primary care records
Source: PLoS One. 2025 Feb 10;20(2):e0314084. doi: 10.1371/journal.pone.0314084 (PMC11809798; doi:10.1371/journal.pone.0314084)
Supplement: S1 Table — (PDF) [file pone.0314084.s003.pdf]

Table S1: Read codes used to describe severe chronic kidney disease.

Medcode	Readcode	Description
11773	7L1A.11	Dialysis for renal failure
20073	7L1A000	Renal dialysis
20196	14V2.00	H/O: renal dialysis
22252	ZV45100	[V]Renal dialysis status
44422	14V2.11	H/O: kidney dialysis
46145	ZV56011	[V]Aftercare involving renal dialysis NOS
28158	TB11.00	Kidney dialysis with complication, without blame
60743	ZV56.00	[V]Aftercare involving intermittent dialysis
66714	TB11.11	Renal dialysis with complication, without blame
69266	TA22000	Failure of sterile precautions during kidney dialysis
69760	ZVu3G00	[X]Other dialysis
96184	TA02000	Accid cut,puncture,perf,h'ge - kidney dialysis
64636	7L1Az00	Compensation for renal failure NOS
31549	7L1A.00	Compensation for renal failure
83513	7L1C.00	Placement other apparatus for compensation for renal failure
56760	7L1B.00	Placement ambulatory apparatus compensation renal failure
48022	7L1Ay00	Other specified compensation for renal failure
65089	7L1Cz00	Placement other apparatus- compensate for renal failure NOS
107901	7L1Cy00	Placement other apparatus- compensate for renal failure OS
52088	ZV56100	[V]Preparatory care for dialysis
52123	8882	Intestinal dialysis
36442	7L1B.11	Placement ambulatory dialysis apparatus - compens renal fail
99692	ZV56000	[V]Aftercare involving extracorporeal dialysis
101912	Z1A..00	Dialysis training
109884	SP0H.00	Disorder associated with dialysis
111637	TA42000	Mechanical failure of apparatus during kidney dialysis
54844	U612200	[X]Failure sterile precautions dur kidney dialys/other perf
63038	ZV56z00	[V]Unspecified aftercare involving intermittent dialysis
63488	ZV56y00	[V]Other specified aftercare involving intermittent dialysis
2994	7L1A100	Peritoneal dialysis
8037	7L1B000	Insertion of ambulatory peritoneal dialysis catheter
23773	7L1B100	Removal of ambulatory peritoneal dialysis catheter
30709	7L1C000	Insertion of temporary peritoneal dialysis catheter
30756	7L1A500	Continuous ambulatory peritoneal dialysis
64828	7L1A600	Peritoneal dialysis NEC
88597	7L1A400	Automated peritoneal dialysis

59194	7L1By00	Placement ambulatory apparatus- compensate renal failure OS
36442	7L1B.11	Placement ambulatory dialysis apparatus - compens renal fail
101124	SP06B00	Continuous ambulatory peritoneal dialysis associated perit
104719	Z1A1.00	Peritoneal dialysis training
107900	SP0E.00	Disorders associated with peritoneal dialysis
110976	Z1A1.11	PD - Peritoneal dialysis training
111103	SP0E100	Thrombus in peritoneal dialysis catheter
45160	ZV56y11	[V]Aftercare involving peritoneal dialysis
46438	SP05613	[X] Peritoneal dialysis associated peritonitis
48639	SP01500	Mechanical complication of dialysis catheter
101756	7L1A011	Thomas intravascular shunt for dialysis
2996	7L1A200	Haemodialysis NEC
60302	7A60600	Creation of graft fistula for dialysis
71124	7L1A300	Haemofiltration
108785	SP0F.00	Haemodialysis first use syndrome
74905	Z1A2.00	Haemodialysis training
59315	SP07G00	Stenosis of arteriovenous dialysis fistula
60446	Z919.00	Care of haemodialysis equipment
60498	Z919300	Reversing haemodialysis lines
72336	Z919100	Priming haemodialysis lines
105436	SP0G.00	Anaphylactoid reaction due to haemodialysis
105742	G72D200	Aneurysm of anastomotic site of dialysis AV fistula
105760	G72C.00	Ruptured aneurysm of dialysis vascular access
106720	Gy21.00	Thrombosis of dialysis arteriovenous fistula
106975	Gy51.00	Haemorrhage of dialysis arteriovenous fistula
107082	Gy31.00	Occlusion of dialysis arteriovenous fistula
107188	G72D.00	Aneurysm of dialysis arteriovenous fistula
107220	G72D100	Aneurysm of needle site of dialysis arteriovenous fistula
107260	Gy41.00	Infection of dialysis arteriovenous fistula
107719	7A61A00	Ligation of arteriovenous dialysis graft
107746	Gy1..00	Stenosis of dialysis vascular access
108116	Gy3..00	Occlusion of dialysis vascular access
108213	Gy40.00	Infection of dialysis arteriovenous graft
108423	Gy60.00	Rupture of dialysis arteriovenous graft
108699	Gy10.00	Stenosis of dialysis arteriovenous graft
108759	Gy5..00	Haemorrhage of dialysis vascular access
109135	Gy30.00	Occlusion of dialysis arteriovenous graft
109809	Gy2..00	Thrombosis of dialysis vascular access
110051	Gy4..00	Infection of dialysis vascular access
110072	Z919200	Washing back through haemodialysis lines
110095	G72D000	Aneurysm of superficialised artery of dialysis AV fistula
12479	1Z13.00	Chronic kidney disease stage 4
12585	1Z14.00	Chronic kidney disease stage 5
95122	1Z1H.00	Chronic kidney disease stage 4 with proteinuria
95405	1Z1L.00	Chronic kidney disease stage 5 without proteinuria

95406	1Z1J.00	Chronic kidney disease stage 4 without proteinuria
104963	K054.00	Chronic kidney disease stage 4
105151	K055.00	Chronic kidney disease stage 5
97587	1Z1J.11	CKD stage 4 without proteinuria
97683	1Z1L.11	CKD stage 5 without proteinuria
99160	1Z1K.11	CKD stage 5 with proteinuria
99312	1Z1H.11	CKD stage 4 with proteinuria
109904	1Z1b.00	CKD with GFR category G4 & albuminuria category A2
109980	1Z1a.00	CKD with GFR category G4 & albuminuria category A1
109981	1Z1e.00	CKD with GFR category G5 & albuminuria category A2
110133	1Z1d.00	CKD with GFR category G5 & albuminuria category A1
110467	1Z1f.00	CKD with GFR category G5 & albuminuria category A3
110626	1Z1c.00	CKD with GFR category G4 & albuminuria category A3
95508	1Z1K.00	Chronic kidney disease stage 5 with proteinuria
2997	7B00.00	Transplantation of kidney
5504	7B00z00	Transplantation of kidney NOS
5911	ZV42000	[V]Kidney transplanted
11553	SP08300	Kidney transplant failure and rejection
11745	7B00100	Transplantation of kidney from live donor
18774	TB00111	Renal transplant with complication, without blame
24361	7B00200	Transplantation of kidney from cadaver
26862	7B06300	Exploration of renal transplant
48057	K0B5.00	Renal tubulo-interstitial disordrs in transplant rejectn
66705	7B00111	Allotransplantation of kidney from live donor
70874	7B00y00	Other specified transplantation of kidney
72004	7B01511	Excision of rejected transplanted kidney
89924	7B00300	Allotransplantation of kidney from cadaver, heart-beating
93366	7B0F.00	Interventions associated with transplantation of kidney
94964	7B0F400	Post-transplantation of kidney examination, live donor
96133	7B00400	Allotransplantation kidney from cadaver, heart non-beating
100693	Kyu1C00	[X]Renal tubulo-interstitial disorders/transplant rejection
54990	TB00100	Kidney transplant with complication, without blame
49028	14S2.00	H/O: kidney recipient
98364	7B00211	Allotransplantation of kidney from cadaver
70712	SP08011	Det.ren.func.after ren.transpl
96095	7B0F200	Pre-transplantation of kidney work-up, live donor
90952	7B0F100	Pre-transplantation of kidney work-up, recipient
48121	7B01500	Transplant nephrectomy
103429	7B0F300	Post-transplantation of kidney examination, recipient
104049	7B0Fz00	Interventions associated with transplantation of kidney NOS
104050	7B0Fy00	OS interventions associated with transplantation of kidney
104201	SP08H00	Acute rejection of renal transplant
104630	SP08G00	Acute rejection of renal transplant - grade III
104905	SP08D00	Acute-on-chronic rejection of renal transplant
104960	SP08E00	Acute rejection of renal transplant - grade I
105328	7B00212	Cadaveric renal transplant

105724	SP08N00	Unexplained episode of renal transplant dysfunction
105811	SP08R00	Renal transplant rejection
106620	SP08J00	Chronic rejection of renal transplant
107000	SP08F00	Acute rejection of renal transplant - grade II
107752	SP08T00	Urological complication of renal transplant
108437	SP08V00	Very mild acute rejection of renal transplant
109455	7B00500	Allotransplantation of kidney from cadaver NEC
106301	SP08P00	Stenosis of vein of transplanted kidney
48022	7L1Ay00	Other specified compensation for renal failure
350	K06..00	Renal failure unspecified
6712	K050.00	End stage renal failure
8330	K0D..00	End-stage renal disease
11554	SP15400	Renal failure as a complication of care
15945	SK05.00	Renal failure following crush syndrome
39598	SP15411	Kidney failure as a complication of care
47342	Q48y000	Congenital renal failure
53852	K05..12	End stage renal failure
53940	Kyu2100	[X]Other chronic renal failure
61930	Kyu2.00	[X]Renal failure
63760	SK05.11	Renal failure after crushing
64636	7L1Az00	Compensation for renal failure NOS
107771	K06..12	Kidney failure unspecified
106860	C353600	Renal failure-associated hyperphosphataemia
512	K05..00	Chronic renal failure
71314	L093.00	Renal failure following abortive pregnancy
12720	1Z1..00	Chronic renal impairment
