# Supplementary material for: Identifying children who develop severe chronic kidney disease using primary care records
Source: PLoS One. 2025 Feb 10;20(2):e0314084. doi: 10.1371/journal.pone.0314084 (PMC11809798; doi:10.1371/journal.pone.0314084)
Supplement: S2 Table — (PDF) [file pone.0314084.s004.pdf]

Table S2: The association of predictive symptoms and a subsequent severe CKD code between cases and controls with no prior evidence of kidney disease, 24 and 6 months prior to index date.

	Controls		Cases	Diagnostic utilities				Conditional regression
	N	%	N	Sensitivity (%)	Specificity (%)	PPV (95% CI) per 1000	LR (95% CI)	Odds Ratio (95% CI)
**24-months before index**								
	1,962		119					2,081
Oedema	-*	-*	-*	-*	99	0.5 (0.2, 1.7)	5.5 (1.9, 16.8)	5.70 (1.82, 17.83)
UTI	59	3	9	8	97	0.3 (0.1, 0.5)	2.5 (1.3, 5.0)	2.73 (1.26, 5.88)
Vision concerns	-*	-*	-*	-*	100	0.5 (0.1, 2.7)	5.5 (1.1, 26.9)	5.15 (1.02, 25.99)
Vomiting	89	5	14	12	96	0.3 (0.2, 0.4)	2.6 (1.5, 4.4)	2.77 (1.51, 5.09)
Growth concerns	-*	-*	-*	-*	100	0.9 (0.3, 3.2)	9.4 (2.8, 31.7)	10.88 (2.82, 41.96)
**6-months before index**								
	2,353		134					2,487
Back pain	-*	-*	-*	-*	99	0.4 (0.1, 1.1)	3.7 (1.3, 10.7)	4.02 (1.29, 12.51)
Generally unwell	-*	-*	-*	-*	100	0.5 (0.1, 2.4)	5.0 (1.1, 23.9)	5.88 (1.16, 29.7)
Oedema	-*	-*	-*	-*	100	1.8 (0.4, 8.5)	17.6 (3.6, 86.2)	18.17 (3.56, 92.69)
UTI	20	0.8	5	4	99	0.4 (0.1, 1.2)	4.4 (1.7, 11.5)	4.13 (1.54, 11.08)
Vomiting	36	2	11	8	99	0.5 (0.3, 1.0)	5.4 (2.8, 10.3)	5.53 (2.74, 11.13)
Growth concerns	-*	-*	-*	-*	100	2.3 (0.5, 10.3)	23.4 (5.3, 103.6)	23.33 (5.2, 104.67)
Urinary symptom (excl. UTI)	-*	-*	-*	-*	99	0.3 (0.1, 0.9)	3.1 (1.1, 8.7)	3.18 (1.08, 9.38)

Abbreviations: CI, confidence interval; LR, positive likelihood ratio; PPV, positive predictive value; UTI, Urinary tract infection.

**Cells containing fewer than 5 cases are suppressed in line with Clinical Practice Research Datalink's policy.*
