# Supplementary material for: Identifying children who develop severe chronic kidney disease using primary care records
Source: PLoS One. 2025 Feb 10;20(2):e0314084. doi: 10.1371/journal.pone.0314084 (PMC11809798; doi:10.1371/journal.pone.0314084)
Supplement: S3 Table — (PDF) [file pone.0314084.s005.pdf]

Table S3: The association of predictive symptoms and a subsequent severe CKD code between cases and controls under 18 years of age, 24 and 6 months prior to index date.

	Controls		Cases	Diagnostic utilities				Conditional regression
	N	%	N	Sensitivity (%)	Specificity (%)	PPV (95% CI)	LR (95% CI)	Odds Ratio (95% CI)
**24 months before index**								
	2,836		174					3,010
Generally unwell	36	1	6	4	99	0.3, 0.1, 0.6)	2.7 (1.2, 6.4)	2.57 (1.10, 6.20)
Growth concerns	9	0.3	8	5	100	1.4 (0.6, 3.7)	14.5 (5.7, 37.1)	15.20 (5.80, 39.5)
Oedema	17	0.6	8	5	99	0.8 (0.3, 1.7)	7.7 (3.4, 17.5)	7.45 (3.20, 17.3)
UTI	78	3	12	7	97	0.3 (0.1, 0.5)	2.5 (1.4, 4.5)	2.74 (1.40, 5.30)
Vomiting	132	5	19	11	95	0.2 (0.1, 0.4)	2.4 (1.5, 3.7)	2.51 (1.50, 4.20)
**6 months before index**								
	3,361		194					3,555
Back pain	-*	-*	-*	-*	99	0.4 (0.1, 1.1)	3.7 (1.3, 10.6)	3.36 (1.12, 10.10)
Generally unwell	-*	-*	-*	-*	100	0.6 (0.2, 2.4)	6.5 (1.7, 24.3)	6.11 (1.60, 23.36)
Growth concerns	-*	-*	-*	-*	100	8.6 (1.0, 68.7)	86.6 (10.2, 737.8)	94.96 (11.09, 813.11)
Headache	44	1	7	4	99	0.3 (0.1, 0.6)	2.8 (1.3, 6.0)	2.87 (1.25, 6.60)
Oedema	-*	-*	-*	-*	100	0.9 (0.2, 4.7)	8.7 (1.6, 47.0)	8.37 (1.52, 46.14)
Vomiting	41	1	9	5	99	0.4 (0.2, 0.8)	3.8 (1.9, 7.7)	3.98 (1.90, 8.32)

Abbreviations: CI, confidence interval; LR, positive likelihood ratio; PPV, positive predictive value; UTI, Urinary tract infection.
**Cells containing fewer than 5 cases are suppressed in line with Clinical Practice Research Datalink's policy.*
