# Supplementary material for: Identifying children who develop severe chronic kidney disease using primary care records
Source: PLoS One. 2025 Feb 10;20(2):e0314084. doi: 10.1371/journal.pone.0314084 (PMC11809798; doi:10.1371/journal.pone.0314084)
Supplement: S4 Table — (PDF) [file pone.0314084.s006.pdf]

Table S4: Test characteristics at ascending cut-off points of clinical risk prediction score for severe CKD.

Varying predicted risk thresholds	N (%) with positive score	Sensitivity (%)	Specificity (%)	PPV (%)
<2%	3,064 (56.6)	52.4%	43.2%	0.009%
>2%	1,480 (27.3)	17.9%	72.1%	0.006%
>5%	504 (9.3)	9.3%	90.7%	0.01%
>10%	312 (5.8)	14.5%	94.7%	0.03%
>15%	56 (1.0)	5.9%	99.2%	0.1%

Abbreviations: PPV, positive predictive value.
