# Supplementary material for: Identifying children who develop severe chronic kidney disease using primary care records
Source: PLoS One. 2025 Feb 10;20(2):e0314084. doi: 10.1371/journal.pone.0314084 (PMC11809798; doi:10.1371/journal.pone.0314084)
Supplement: S1 File — (PDF) [file pone.0314084.s007.pdf]

File S1: Symptom/sign exposure variable codelists

Codelists for each symptom/sign exposure examined were developed using a systematic approach as described by Watson *et al* https://bmjopen.bmj.com/content/bmjopen/7/11/e019637.full.pdf). Firstly, symptoms and signs considered to be suggestive of chronic kidney disease were compiled from a number of sources, including feedback from patient groups, children and young people with advanced kidney disease and their families and the paediatric and adult literature. Terms and definitions for each symptom variable were collated from several clinical sources:

- the International Classification of Primary Care (ICPC), 2nd version
- Medical Subject Headings (MeSH)
- the National Institute for Health and Care Excellence (NICE) Clinical Knowledge Summaries
- The International Classification of diseases, 10th revision (ICD-10).

Each variable and its defining terms were subsequently reviewed by an academic GP (Professor Matthew Ridd) to ensure appropriate terms were included or excluded, respectively. Secondly, all potential Read codes relating to each symptom variable were identified from the CPRD medical dictionary. This file was searched for codes relating to the defining terms for each symptom variable. To ensure a systematic, reproducible approach, the latest version of Stata available from the University of Bristol was used to search the medical file, using the regular expression lookup (*regexm*) command. For each symptom variable, identified medcodes were exported to an Excel file for manual review. Where possible, pre-existing, validated code lists from previous CPRD paediatric research held by the University of Bristol were used to supplement code lists, with duplicates removed.

Finally, each code list was independently reviewed by the author of this thesis and a practicing paediatric nephrologist (Dr Manish Sinha). Each reviewer categorised each search term using a three-point scale, with comments added where necessary:

1. Definitely exclude: the code does not represent the variable of interest.
2. Uncertain.
3. Definitely include: the code accurately represents the variable of interest.

Where discrepancy among reviewers existed for individual codes, discussion led to resolution and a final, agreed code was entered. The final symptom/sign codelists used to determine exposure status are publicly available at: https://github.com/drlucyplumb/Identifyingsevereckd/blob/main/Identifying%20children%20with%20severe%20CKD%20using%20primary%20care%20records-symptom%20library.xlsx
