# Supplementary figures and images for: Identifying children who develop severe chronic kidney disease using primary care records
Source: PLoS One. 2025 Feb 10;20(2):e0314084. doi: 10.1371/journal.pone.0314084 (PMC11809798; doi:10.1371/journal.pone.0314084)

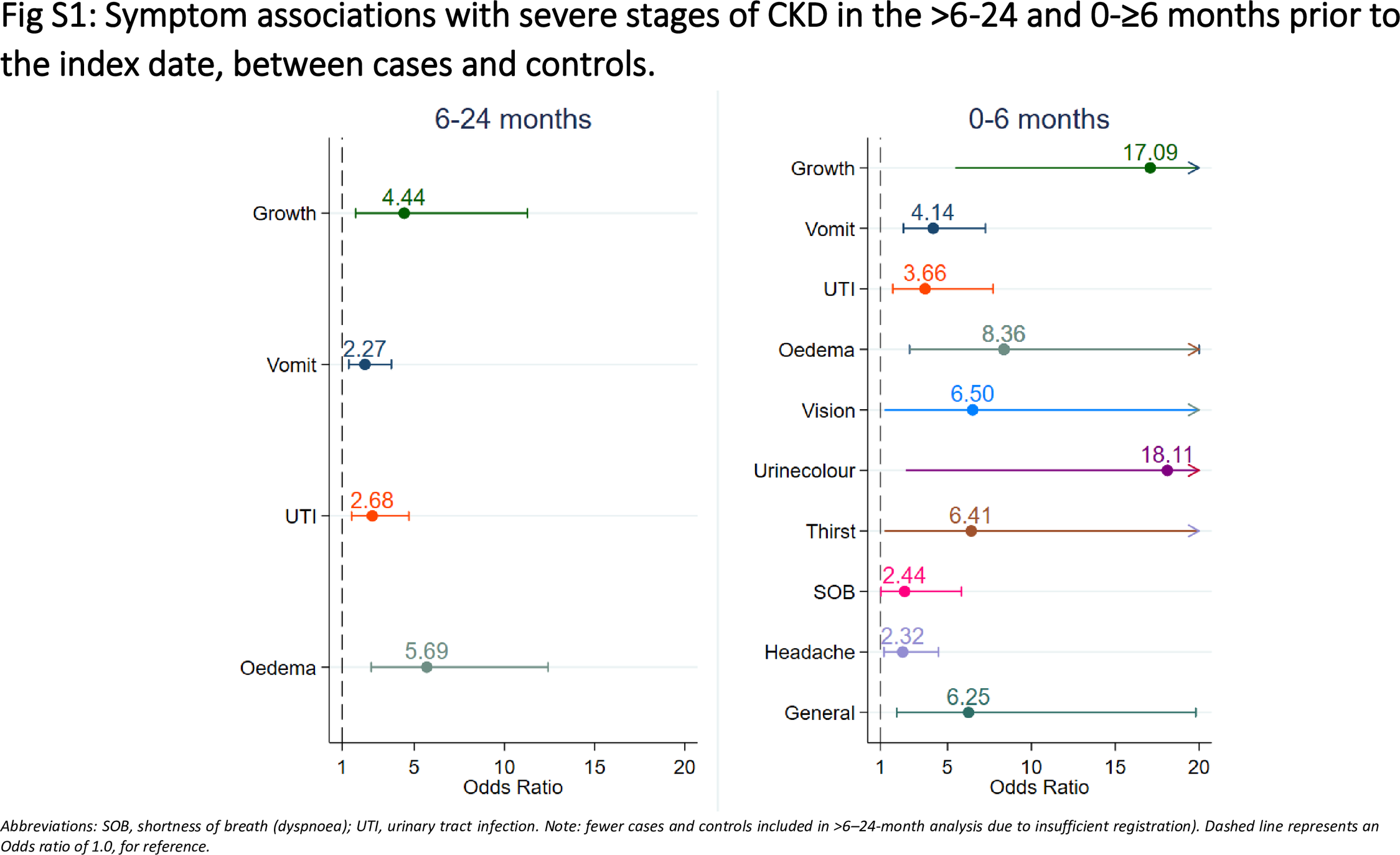

Supplement: S1 Fig — (TIF) [file pone.0314084.s008.tif]

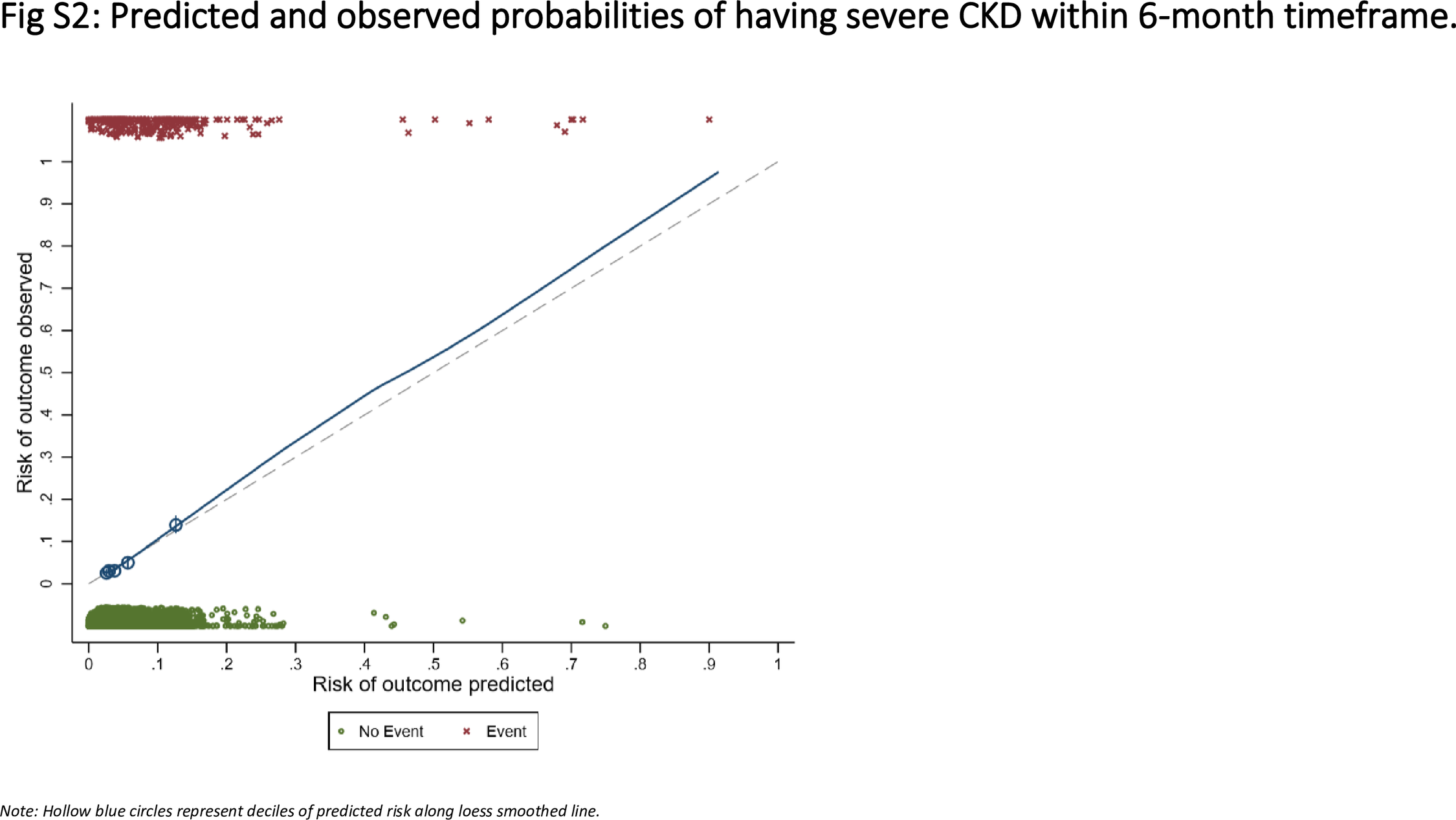

Supplement: S2 Fig — (TIF) [file pone.0314084.s009.tif]
