# Supplementary material for: Identifying children who develop severe chronic kidney disease using primary care records
Source: PLoS One. 2025 Feb 10;20(2):e0314084. doi: 10.1371/journal.pone.0314084 (PMC11809798; doi:10.1371/journal.pone.0314084)
Supplement: Attachment — Submitted filename: Review assignment for PONE-D-24-05195 20may2024.docx [file pone.0314084.s010.docx]

Here are my comments for review assignment for PONE-D-24-05195,

In general, the manuscript seems to be well written and the statistical analyses applicable.

With exception of Table 2 and row 240, it is a bit unclear if the PPV is calculated for each type of symptoms or for combination of symptoms.

Row 14 - On the conclusion “positive predictive value of symptoms is low”, is the conclusion rather that “In combination, symptoms and consultation frequency demonstrated modest discrimination for CKD”?

Row 135 –  On “there were 340 CYP with evidence of an incident severe CKD code in the primary care record, which were matched to 6,800 controls”, it would be more interesting with the numbers of CYP and controls that was used in the 24 months analysis and the 6 months analysis respectively.

Row 139 – Rater present the demographics table for the subjects included in the 24 months analysis and the 6 months analysis respectively.

Row - 163 Table 2, The heading “PPV per 1000” may be hard to understand, perhaps add a footnote of type ‘PPV 1.8 per 1000’ implies of 1,000 children coded, 2 would be diagnosed with severe CKD”.

Figure 1 – For the “6-month analyses” in panel a and b, should the text “registration less than 12 months” rather be “registration less than 6 months”?
