# Supplementary material for: Identifying children who develop severe chronic kidney disease using primary care records
Source: PLoS One. 2025 Feb 10;20(2):e0314084. doi: 10.1371/journal.pone.0314084 (PMC11809798; doi:10.1371/journal.pone.0314084)
Supplement: Attachment — Submitted filename: Response to reviewers .docx [file pone.0314084.s011.docx]

We thank the reviewers for the insightful and constructive comments. We hope to have addressed the comments satisfactorily.

Review Comments to the Author

Please use the space provided to explain your answers to the questions above. You may also include additional comments for the author, including concerns about dual publication, research ethics, or publication ethics. (Please upload your review as an attachment if it exceeds 20,000 characters)

Reviewer #1: Here are my comments,

With exception of Table 2 and row 240, it is a bit unclear if the PPV is calculated for each type of symptoms or for combination of symptoms.

Thank you for this comment. We have now made clear in the text where we refer to individual symptoms; we have also highlighted that in the prediction model there is mutual adjustment for the included variables.

Row 14 - On the conclusion “positive predictive value of symptoms is low”, is the conclusion rather that “In combination, symptoms and consultation frequency demonstrated modest discrimination for CKD”?

Thank you for this comment. We believe both statements to be accurate, as evidenced by the individual symptoms and their associations with case status (table 2), and as evidenced by the C-statistic in table 4 for symptoms and consultation frequency combined.

Row 135 – On “there were 340 CYP with evidence of an incident severe CKD code in the primary care record, which were matched to 6,800 controls”, it would be more interesting with the numbers of CYP and controls that was used in the 24 months analysis and the 6 months analysis respectively.

We have now amended this section to refer directly to the study cohort used in the two analyses (24 months and 6 months, respectively).

Row 139 – Rater present the demographics table for the subjects included in the 24 months analysis and the 6 months analysis respectively.

Please find table one has now been amended to reflect the study cohorts used.

Row - 163 Table 2, The heading “PPV per 1000” may be hard to understand, perhaps add a footnote of type ‘PPV 1.8 per 1000’ implies of 1,000 children coded, 2 would be diagnosed with severe CKD”.

Thank you for this suggestion. We have added this as a footnote for ease of interpretation.

Figure 1 – For the “6-month analyses” in panel a and b, should the text “registration less than 12 months” rather be “registration less than 6 months”?

As age was derived in whole years, children under one on the index date were excluded from the 6-month analysis. This is also referenced in the text (lines 144-145).

Reviewer #2: It is a very relevant topic as it would begin monitoring & reversing some of the factors that can be reversed in chronic renal failure e.g. treatment of urinary tract infection diagnosis and management of hypertension, dietary manipulation, management of short stature and failure to thrive and delaying the onset of renal replacement therapy in theses chronic conditions.
To simplify the scoring system. I recognize that this was a study but for the general practitioners & general paediatricians, we need a simple algorithm.

Thank you for this suggestion. We present the risk score as a footnote to table 4. We are planning to externally validate this algorithm before developing a risk calculator for ease of use clinically.

It is a very relevant study.
